# Brain Age Modeling and Cognitive Outcomes in Young Adults With and Without Sickle Cell Anemia

**DOI:** 10.1001/jamanetworkopen.2024.53669

**Published:** 2025-01-17

**Authors:** Andria L. Ford, Slim Fellah, Yan Wang, Kira Unger-Levinson, Maria Hagan, Martin N. Reis, Amy Mirro, Josiah B. Lewis, Chunwei Ying, Kristin P. Guilliams, Melanie E. Fields, Hongyu An, Allison A. King, Yasheng Chen

**Affiliations:** 1 Department of Neurology, Washington University in St Louis School of Medicine, St Louis, Missouri; 2 Mallinckrodt Institute of Radiology, Washington University in St Louis School of Medicine, St Louis, Missouri; 3 Department of Pediatrics, Washington University in St Louis School of Medicine, St Louis, Missouri; 4 Program in Occupational Therapy, Washington University in St Louis School of Medicine, St Louis, Missouri

## Abstract

**Importance:**

Both sickle cell anemia (SCA) and socioeconomic status have been associated with altered brain structure and cognitive disability, yet precise mechanisms underlying these associations are unclear.

**Objective:**

To determine whether brains of individuals with and without SCA appear older than chronological age and if brain age modeling using brain age gap (BAG) can estimate cognitive outcomes and mediate the association of socioeconomic status and disease with these outcomes.

**Design, Setting, and Participants:**

In this cross-sectional study of 230 adults with and without SCA, individuals underwent brain magnetic resonance imaging (MRI) and cognitive assessment. Brain age was estimated using DeepBrainNet, a model trained to estimate chronological age from 14 468 structural MRIs from healthy individuals across the lifespan. BAG was defined as estimated brain age minus chronological age. Linear regression examined clinical factors associated with BAG and the ability of BAG to estimate cognitive performance compared to neuroimaging metrics of brain health and ischemic brain injury, such as normalized whole brain volume, white matter mean diffusivity (MD), and infarct volume. BAG and white matter MD were tested further as mediators of the association of socioeconomic status and SCA with cognitive performance. Data were analyzed from October 15, 2023, to July 1, 2024.

**Exposures:**

SCA disease status and economic deprivation as measured using the area deprivation index (ADI).

**Main Outcome and Measures:**

Executive function, crystallized function, processing speed, and full-scale intelligence quotient (FSIQ) were derived from the National Institutes of Health (NIH) Toolbox and Wechsler Abbreviated Scale of Intelligence, Second Edition.

**Results:**

Among 230 included adults, 123 individuals had SCA (median [IQR] age, 26.4 [21.8-34.3] years; 77 female [63%]) and 107 individuals did not (control cohort; median [IQR] age, 30.1 [26.3-34.8] years; 77 female [72%]). Participants with SCA had a larger median (IQR) BAG compared to individuals in the control cohort (14.2 [8.0-19.2] vs 7.3 [3.2-11.1] years; median difference, 6.13 years; 95% CI, 4.29-8.05 years; *P* < .001). Individuals in the control cohort demonstrated a larger BAG relative to the reference population (mean difference, 7.52 years; 95% CI, 6.32-8.72 years; *P* < .001). Higher economic deprivation was associated with BAG in the control cohort (β [SE] per 1% ADI increase, 0.079 [0.028]; 95% CI, 0.023 to 0.135; *P* = .006), while intracranial vasculopathy (β [SE], 6.562 [1.883]; 95% CI, 2.828 to 10.296; *P* < .001) and hemoglobin S percentage (β [SE] per 1% increase, 0.089 [0.032]; 95% CI, 0.026 to 0.151; *P* = .006) were associated with BAG in participants with SCA. Across neuroimaging metrics of brain health, BAG demonstrated the largest effect size for cognitive outcomes in the control cohort (eg, executive function: *r* = −0.430; *P* = .001), while white matter MD demonstrated the largest effect size for cognitive outcomes (eg, executive function: *r* = −0.365; *P* = .001) in the SCA cohort. Across the study population, BAG mediated the association of ADI with cognitive performance (eg, executive function: β [SE] per 1-unit decrease in ADI, −0.031 [0.014]; 95% CI, −0.061 to −0.006), while BAG (eg, FSIQ: β [SE], −3.79 [1.42]; 95% CI, −6.87 to −1.40) and white matter MD (eg, FSIQ: β [SE], −4.55 [1.82]; 95% CI, −8.14 to −0.94) mediated the association of SCA with cognitive performance.

**Conclusions and Relevance:**

Adults with SCA and a healthy control cohort with greater economic deprivation demonstrated older brain age, suggestive of insufficient brain development, premature brain aging, or both. Brain estimates of chronological age may inform mechanisms of the association between chronic disease and socioeconomic status with cognitive outcomes in healthy and SCA populations, yet will require confirmation in larger and longitudinal studies.

## Key Points

**Question:**

Do brain magnetic resonance imaging estimates of age using brain age modeling inform mechanisms underlying cognitive impairment in adults with and without sickle cell anemia (SCA)?

**Findings:**

In this cross-sectional study of 230 adults with and without SCA, brain age gap was elevated in individuals with vs without SCA and was elevated in those without SCA relative to the DeepBrainNet reference population. Brain age gap mediated associations of economic deprivation and disease with cognitive outcomes in individuals with and without SCA.

**Meaning:**

Estimates of brain age may inform mechanisms underlying the association of chronic disease and socioeconomic status with cognition in both healthy and SCA populations.

## Teaser

This cross-sectional study investigates the association of brain age estimates with cognitive outcomes in young adults with and without sickle cell anemia.

## Introduction

Individuals with sickle cell anemia (SCA) carry a lifelong risk of cerebral hypoxic-ischemic injury and associated cognitive disability.^1,2^ The critical negative impact on children has been recognized primarily in domains of executive function and processing speed, with decreased ability to meet academic milestones.^3^ Moreover, adults with SCA are unable to catch up to their peers without SCA. Rather, the gap in cognitive performance widens with age, amplifying cognitive consequences over time.^4^ Cognitive impairment occurs even in the absence of stroke, resulting in challenges with school and maintaining employment.^5,6,7^ While initially focused on SCA, results of prior studies have unexpectedly revealed the association of socioeconomic status and parental education with cognitive outcomes independent of and beyond the presence of overt and silent strokes in SCA cohorts.^8^ Furthermore, studies in healthy pediatric populations have demonstrated the association of socioeconomic status and poverty with brain development (the income-to-needs ratio was operationalized as the total family income divided by the federal poverty level based on family size in the year most proximal to data collection), while there are fewer reports of how socioeconomic status impacts brain aging in healthy adults.^9,10,11^

Structural and functional brain magnetic resonance imaging (MRI) methods provide biomarkers of brain development and cerebral ischemic vulnerability to understand to what extent the brain changes as individuals grow and age.^1,12,13^ Gray and white matter show delayed growth trajectories in children with SCA,^14^ as well as regional brain atrophy in the setting of intracranial vasculopathy or Moyamoya syndrome.^15^ White matter microstructural changes are apparent beyond regions of cerebral infarction in adults with SCA.^16^ While several studies have correlated brain imaging with cognitive outcomes in children with SCA, few studies have been performed in adults, which may be increasingly important as individuals live longer with SCA.^14,17,18,19^

Recently, studies estimating chronological age based on structural brain MRI and calculation of a brain age gap (BAG) in healthy and disease states have increased to determine whether BAG provides a biomarker associated with brain health and disease severity. Toward this end, deep learning methods have been applied to large training datasets with a goal to predict the chronological age of healthy brains.^20,21^ Models have since been applied to chronic diseases, such as Alzheimer dementia and schizophrenia, to determine their estimative nature for disease progression.^22,23^ The DeepBrainNet model utilized a publicly available convolutional neural network to train T1 MRI scans from 14 468 healthy individuals, enabling brain age estimation across the lifespan.^24^ Accurate modeling of a large control cohort, such as in DeepBrainNet, permits comparison of BAG in disease and control cohorts to determine if they differ from the reference population.

With advances in newborn screening and transcranial Doppler ultrasound for stroke prevention, individuals with SCA are living longer, while the association of SCA with brain aging and cognitive impairment is understudied.^25,26^ We hypothesized that the downstream outcomes of chronic hypoxia-ischemia in SCA may not only slow brain development, but also accelerate brain aging, such that BAG would be increased and could be leveraged as a biomarker for brain health and cognitive outcomes. Secondarily, we hypothesized that economic deprivation may be associated with older brain ages in control and SCA cohorts, such that BAG would mediate the association of socioeconomic status and disease with cognition.

## Methods

### Participants

The Institutional Review Board of Washington University School of Medicine approved this cross-sectional study. Written informed consent was obtained from all participants. The authors adhered to the Strengthening the Reporting of Observational Studies in Epidemiology (STROBE) reporting guideline for observational studies.

Healthy adults and adults with SCA were prospectively enrolled in a cross-sectional study between 2017 and 2023 in St Louis City and the surrounding St Louis County region in eastern Missouri and southwestern Illinois. Inclusion criteria for the SCA cohort were individuals aged 18 years or older with hemoglobin (Hb) S β^0^ thal or HbS β^+^ thal or HbSC. Individuals receiving long-term exchange transfusions, with a history of overt or silent stroke, or history of intracranial vasculopathy were included. Participants in the control cohort self-identified as healthy and Black race. (Given that most patients with SCD are Black in the US, patients in the control cohort were matched to the race of the SCD cohort.) Exclusion criteria for the SCA cohort were any contraindication to MRI, pregnant or breastfeeding, history of stem cell transplantation, and history of revascularization surgery for Moyamoya. Additional exclusion criteria for the control cohort were history of neurological disease or any cardiovascular risk factors requiring medication. The control cohort was recruited from 2 sources: an SCA outpatient clinic where partners, friends, and siblings of patients were invited to participate and advertisements posted in the hospital and medical center requesting volunteers for participation.

Stroke history was classified as: (1) overt stroke, defined as a clinical history of stroke associated with acute symptoms, or (2) silent cerebral infarcts (SCIs), defined as hyperintense lesions 3 mm or greater in diameter on axial plane of fluid-attenuated inversion recovery (FLAIR) images, as adjudicated by a board-certified neuroradiologist (M.N.R.).^27^ Demographics, medical history, medications, and vital signs were collected at each scan visit. Peripheral blood sampling measured Hb and capillary gel electrophoresis to confirm the Hb genotype. The area deprivation index (ADI), as an indicator of socioeconomic status, was obtained by entering the participant address into the ADI calculator developed at the University of Wisconsin-Madison Center for Health Disparities.^28,29^ This is a cross-sectional analysis of an observational, longitudinal MRI-cognitive study with a 3 year follow-up. During study start in 2017, only brain MRIs were obtained. When study funding was obtained in 2020, cognitive assessment was added to be performed on the same day as the brain MRI. Participants who enrolled between 2017 and 2020 were invited back for follow-up visits, which then included cognitive testing. For participants with multiple time points, the first time point that included both brain MRI and cognitive assessment was used. Only 1 study time point was included per participant. The current analysis was cross-sectional instead of longitudinal as the majority of participants had not yet returned for follow-up.

### Imaging Protocol

#### MRI Acquisition, Segmentation, and Coregistration

Participants underwent brain MRI and time-of-flight magnetic resonance angiography (TOF MRA) on a Siemens 3 Tesla MR system (Siemens Healthineers). Standard 3-dimensional magnetization prepared rapid gradient echo (MPRAGE) T1-weighted (echo time/repetition time [TE/TR] = 2.95/1800 ms; TI = 1000 ms; flip angle = 8°; resolution = 1.0 × 1.0 ×1.0 mm) and 2-dimensional T2-weighted FLAIR (TE/TR = 93/9000 ms; TI = 2500 ms; resolution = 1.0 × 0.9 × 3.0 mm) were acquired. Parameters for 3-dimensional TOF MRA were TE/TR = 3.59/21.0 ms, TD = 0 ms, flip angle = 18°, and resolution = 0.6 × 0.6 × 0.7 mm. T1 MPRAGE images were skull-stripped and segmented into gray and white matter using Statistical Parametric Mapping software version 12 (SPM12).^30^ Coregistration aligned images within a scan using the Functional Magnetic Resonance Imaging of the Brain (FMRIB) Software Library Linear Image Registration Tool, with accuracy confirmed by visual inspection. White and gray matter volumes were added to calculate whole brain volume, which was normalized to total intracranial volume to adjust for head size. Given that T1 MPRAGE is used as the primary image for age estimation and may be impacted by artifacts, such as motion, we calculated FreeSurfer Euler number, a metric of image quality, using FreeSurfer software version 7.2 (Martinos Center for Biomedical Imaging).^31^

A board-certified neuroradiologist (M.N.R.), blinded to participant cohort, reviewed all MRIs for SCIs, as defined previously,^27^ and MRAs for vasculopathy. The intracranial carotid artery and the first segments of the anterior, middle, and posterior cerebral arteries and basilar artery were examined for the presence of vasculopathy, which was defined as a narrowing of 50% or more.^32^ For SCIs identified by the neuroradiologist, infarcts were manually delineated on the FLAIR map using Medical Image Processing, Analysis and Visualization software version 8.3 (National Institutes of Health [NIH] Center for Information Technology)^33^ by a board-certified vascular neurologist (A.L.F.) blinded to participant cohort.

#### Diffusion Tensor Imaging

Diffusion tensor imaging (DTI; TE/TR = 89/10 100 ms; resolution = 2.0 × 2.0 × 2.0 mm; 25 directions; *b* = 0-1400 s/mm^2^) was processed using the FMRIB Software Library.^34^ Diffusion images were corrected for eddy current distortion and head motion using the *b* = 0 image as a reference. A binary brain mask in DTI space was calculated using the FSL Brain Extraction Tool on the *b* = 0 image. Dtifit^35^ was applied to fit the diffusion tensor model, generating mean diffusivity (MD) at each voxel.

#### Brain Age Gap Measurement

DeepBrainNet was developed for age prediction from a multisite cohort with 14 468 brain T1 images across the lifespan (ages 3-95 years). Of 10 datasets contributing adults to DeepBrainNet, 8 datasets reported race composition, which was reported to be 11% Asian, multiracial, or unknown race; 22% Black; and 67% White. Socioeconomic status was not reported.^24^ The DeepBrainNet deep learning model is based on Inception-resnet-V2 structure with 2 major building elements, inception modules and residual connections, with modification of the established network structure to extract aging features from T1 images with knowledge of participant chronological age. BAG was computed as the difference between the estimated age using the brain T1 map and participant chronological age.^21,36^ A positive BAG indicates that the estimated brain age is older than the chronological age, while a negative BAG indicates a younger estimated age.

### Cognitive Assessment

Cognitive assessment included the NIH Toolbox version 2 (NIH)^37^ and the Wechsler Abbreviated Scale of Intelligence, Second Edition (WASI-2). Each NIH Toolbox test calculates a fully corrected *T* score (mean [SD] 50 [10]), which is corrected for age, sex, race, ethnicity, and level of education. We included 4 tests to determine whether BAG or other neuroimaging metrics were associated with the following cognitive outcomes: executive composite, crystallized composite, processing speed, and full-scale intelligence quotient (FSIQ) composite scores. The executive composite score was computed as the mean of the fully corrected *T* scores for the Dimensional Change Card Sort Test, Flanker Inhibitory Control and Attention Test, and List Sorting Test.^38^ The crystallized composite score was computed as the mean of the fully corrected *T* scores for the Picture Vocabulary Test, Oral Reading Recognition Test, and Picture Sequence Memory Test. Processing speed was measured using the Pattern Comparison Processing Speed fully corrected *T* score. The FSIQ composite score was the sum of the WASI-2 verbal comprehension and matrix reasoning *T* scores.

### Statistical Analysis

Baseline characteristics, laboratory values, and imaging parameters were compared between participants with and without SCA. Mann-Whitney *U* (2 groups) or Kruskal-Wallis (≥3 groups with *H* value and degrees of freedom [*df*] reported) and Fisher exact tests were used for continuous and categorical variables, respectively. The independent-sample Hodges-Lehman median difference provided median differences and 95% CIs. To test BAG in the control vs reference population, a 1-sample *t* test was performed.^39^ To account for multiple testing, significance was adjusted to maintain a family-wise error rate less than 5% using the Benjamini-Hochberg procedure.

For understanding clinical factors associated with BAG, univariate (Pearson *r*) and 3-multivariable linear regression models were examined in total, control, and SCA cohorts. Covariates and criteria for entry are in the eMethods in Supplement 1. Given that BAG was found to be biased toward increasing with younger ages, all models were adjusted for age regardless of strength of association on univariate analysis.^40^ To adjust for image quality and motion, all models were adjusted for Euler number regardless of its association with BAG on univariate analysis. Unstandardized parameter estimates (β), standard error (SE), and 95% CIs are reported. Model fit was confirmed by assessing the distribution of regression residuals. Lack of collinearity was verified with a variance inflation factor less than 2.^41^ Similar regression methods as described previously were used to determine whether BAG or other neuroimaging metrics of brain health, including normalized brain volume, white matter MD, and infarct volume, were independently associated with the following cognitive outcomes: executive composite, crystallized composite, processing speed, and FSIQ composite scores. Infarct volume was not normally distributed and was coded as an ordinal variable in tertiles due to positive skew: 0 to 0.001 cc, 0.001 to 0.3 cc, and more than 0.3 cc.

To examine the hypothesized role of BAG and other neuroimaging metrics as mediators of cognitive outcomes, mediation analyses were performed between ADI and cognition and between SCA and cognition through BAG and white matter MD. Mediators were tested by calculating bias-corrected 95% CIs using bootstrapping with 5000 resamples via the PROCESS procedure for SPSS.^42^

Statistics were conducted using SPSS statistical software version 28.0 (IBM) and GraphPad Prism software version 10 (GraphPad Software, Inc). A 2-sided *P* < .05 indicated statistical significance. Data were analyzed from October 2023 to July 2024.

## Results

A total of 230 participants, including 123 individuals with SCA (median [IQR] chronological age, 26.4 [21.8-34.3] years; 77 female [63%]; 100% Black) and 107 individuals in the control cohort (median [IQR] chronological age, 30.1 [26.3-34.8] years; 77 female [72%]; 100% Black), underwent prospective enrollment with brain MRI (Table 1**)**. A subset of 147 participants underwent MRI and cognitive assessment (eTable 1 in Supplement 1).

**Table 1.** Baseline Participant Characteristics

Characteristic	Participants, No. (%) (N = 230)		*P* value
	Control cohort (n = 107)	SCA cohort (n = 123)	
Age, median (IQR), y^a^	30.1 (26.3-34.8)	26.4 (21.8-34.3)	.005
Estimated brain age, median (IQR), y^b^	39.0 (31.2-44.8)	43.1 (33.8-49.4)	.003
BAG, y^c^	7.3 (3.2-11.1)	14.2 (8.0-19.2)	<.001
Black race	107 (100)	123 (100)	>.99
Sex			
Male	30 (28)	46 (37)	.13
Female	77 (72)	77 (63)	
National ADI, median (IQR), percentile^d^	78 (52-97)	84 (59-96)	.15
Genotype			
HbAA	77 (73)	NA	<.001
HbAS	30 (27)	NA	
HbSS	NA	90 (73)	
HbS β^0^ thal	NA	7 (6)	
HbSC	NA	21 (17)	
HbS β^+^ thal	NA	5 (4)	
Primary disease modification			
Chronic transfusion therapy	NA	24 (20)	NA
Hydroxyurea	NA	48 (39)	NA
Voxelotor	NA	3 (2)	NA
Crizanlizumab	NA	6 (5)	NA
Neurologic status			
Overt stroke	0	17 (14)	<.001
Silent cerebral infarct	44 (41)	69 (56)	.02
Cerebral vasculopathy ≥50%^e^	1/93 (1)	17/116 (15)	<.001
Blood characteristics			
Hemoglobin, g/dL	12.5 (11.9-13.2)	8.7 (7.7-9.8)	<.001
Hematocrit, %	38.2 (35.6-39.7)	25.5 (22.4-28.8)	<.001
Hb S, %	0.0 (0.0-34.1)	71.4 (50.5-81.6)	<.001
Hb F, %	0.0 (0.0-0.0)	5.1 (1.3-15.2)	<.001
Vital signs			
Systolic blood pressure, mm Hg	122 (112-132)	115 (108-124)	.001
Diastolic blood pressure, mmHg	76 (70-83)	70 (65-75)	<.001
SpO_2_, %	99 (98-100)	98 (96-99)	<.001

Abbreviations: ADI, Area Deprivation Index; BAG, brain age gap; Hb, hemoglobin; NA, not applicable; SCA, sickle cell anemia; SpO_2_, oxygen saturation measured by pulse oximetry.

SI conversion factors: To convert hematocrit to proportion of 1.0, multiply by 0.01; hemoglobin to grams per liter, multiply by 10.0.

^a^ Chronological age in years on the day of the magnetic resonance imaging (MRI) scan is shown.

^b^ Estimated participant age brain on T1 and the DeepBrainNet model is shown.

^c^ BAG = estimated brain age – chronological age.

^d^ National ADI percentile (1-100) is given, with higher values demonstrating greater deprivation and lower socioeconomic status based on participant residential address.

^e^ Cerebral vasculopathy was defined as a more than 50% narrowing of the distal internal carotid artery or proximal middle cerebral artery by a board-certified neuroradiologist review of the time-of-flight magnetic resonance angiography performed at the time of the brain MRI scan. The denominator is shown to indicate the number of MRI scans that included magnetic resonance angiography.

### Brain Age Gap in SCA and Control Groups

**Figure 1.**
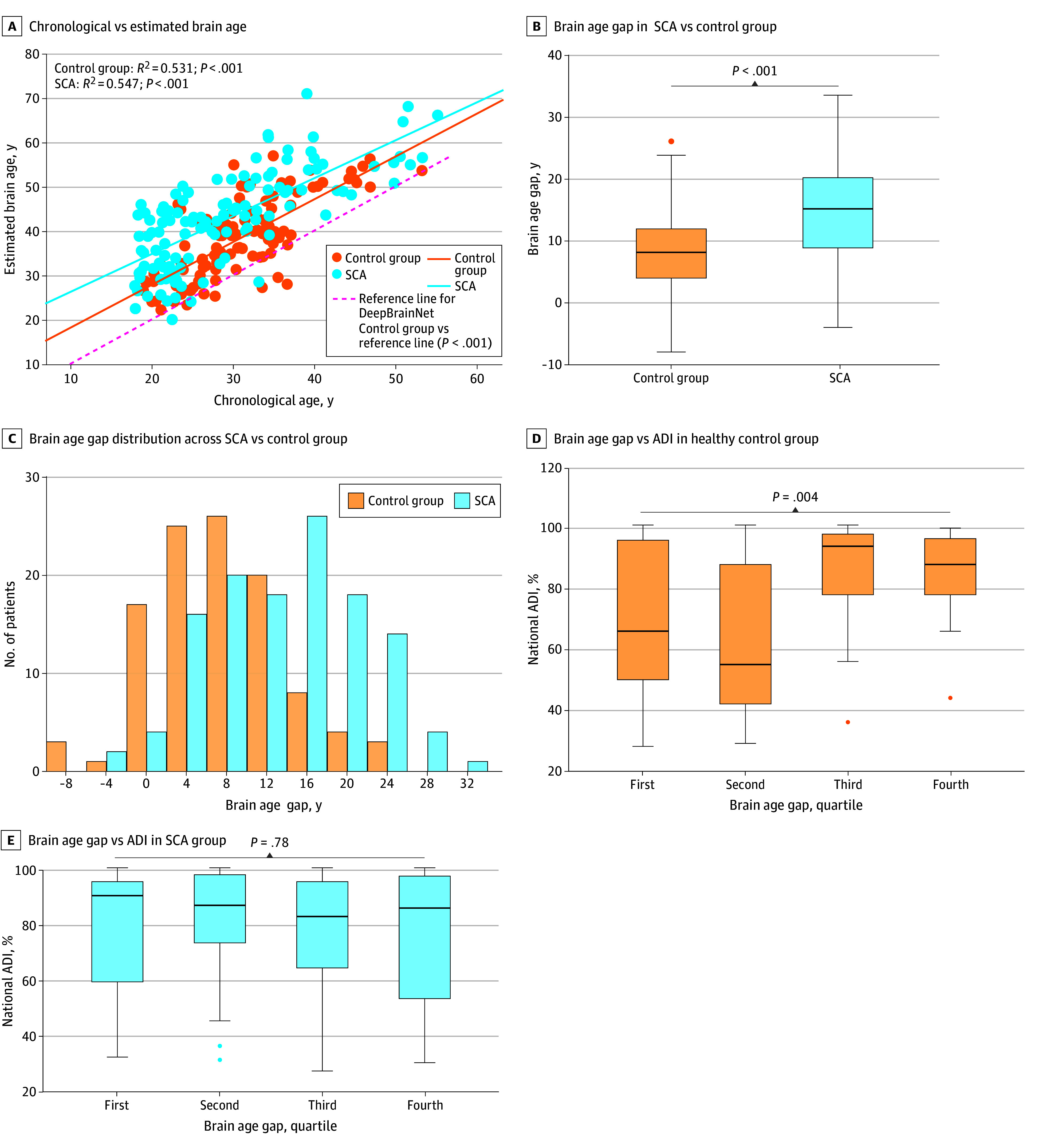
Brain Age Gap in Sickle Cell Anemia (SCA), Control, and Reference Populations A, Estimated brain age correlated with chronological brain age for participants in SCA and control cohorts. The red dashed line indicates the line of identity where the DeepBrainNet reference testing cohort lies. B and C, The brain age gap (estimated brain age – chronological brain age) was higher in the SCA than the control cohort, with histogram distributions shown in C. D and E, Area deprivation index (ADI) was plotted across the 4 quartiles of BAG for the 2 cohorts.

Chronological age correlated with estimated brain age for control (*R*^2^ = 0.531; *P* < .001) and SCA (*R*^2^ = 0.547; *P* < .001) cohorts. Despite a younger chronological age of the SCA vs control cohort (median difference, 2.91 years; 95% CI, 0.99-4.87 years; *P* = .005), the median (IQR) estimated brain age was older (43.1 [(33.8-49.4] vs 39.0 [31.2-44.8] years; median difference, 4.12 years; 95% CI, 1.50-6.85; *P* = .003) and median (IQR) BAG was higher (14.2 [8.0-19.2] vs 7.3 [3.2-11.1] years; median difference, 6.13 years; 95% CI, 4.29-8.05 years; *P* < .001) in the SCA vs control cohort (Table 1; Figure 1A-C). To determine whether increased BAG in SCA was due to inclusion of patients with a history of stroke, vasculopathy, or large SCI volume (>5 cc), 34 such patients were excluded. In this subanalysis, median (IQR) BAG continued to be larger in the SCA vs the control cohort (12.7 [8.0-19.3] vs 7.3 [3.0-11.1] years; median difference, 5.50 years; 95% CI, 3.64-7.52; *P* < .001).

**Figure 2.**
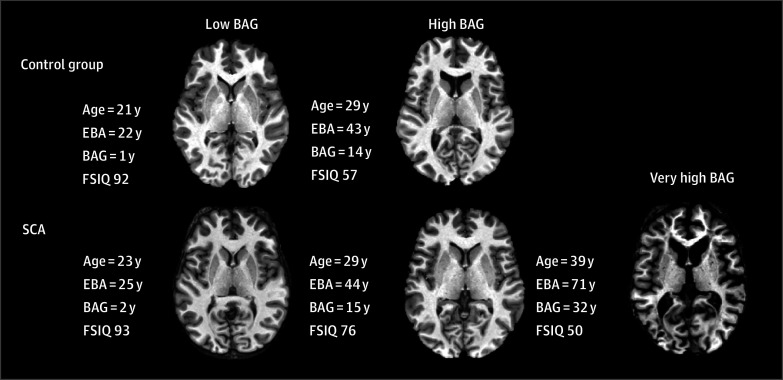
Representative T1 Maps From Which Brain Age Gap (BAG) Was Derived Representative T1 maps of example healthy participants in the control cohort and participants with sickle cell anemia (SCA) qualitatively indicate increased cerebrospinal fluid space and decreased cortical thickness with increasing levels of BAG (low vs high), although T1 features used for brain age calculation are not precisely known. There were no participants in the control cohort with very high BAG. In the SCA cohort, the maximum estimated brain age was 32 years beyond patient chronological age. EBA indicates estimated brain age; FSIQ, full-scale intelligence quotient.

BAG was unexpectedly elevated in the control cohort relative to the reference DeepBrainNet population (mean difference, 7.52 years; 95% CI, 6.32-8.72 years; *P* < .001) (Figure 1A). Representative T1 maps of control and SCA cohorts with low vs high vs very high BAG permit qualitative assessment, although features used for brain age calculation are imprecisely known (Figure 2).

### Clinical Factors Associated With BAG

To understand factors associated with brain growth and aging, linear regression assessed clinical factors associated with BAG in total, SCA, and control cohorts. Across the total cohort, younger age, SCA diagnosis, higher economic deprivation (ADI), and poor image quality (Euler number) correlated with larger BAG. In multivariable analysis, SCA diagnosis (β [SE], 5.930 [0.926]; 95% CI, 4.105 to 7.755; *P* < .001) and Euler number (β [SE] per 1-unit decrease, −0.088 (0.027); 95% CI, −0.141 to −0.035; *P* = .001) remained factors associated with BAG (eTable 2 in Supplement 1).

In the control cohort, ADI, lower hemoglobin, and Euler number were correlated with BAG. Other variables, such as age and sickle cell trait, were not. In multivariable analysis, ADI (β [SE] per 1% increase, 0.079 [0.028]; 95% CI, 0.023 to 0.135; *P* = .006) and lower hemoglobin (β [SE] per 1-g/dL decrease, −0.962 [0.420]; 95% CI −1.796 to −0.128; *P* = .02) were retained in association with BAG (eTable 2 in Supplement 1). In the SCA cohort, intracranial vasculopathy, HbS percentage, age, and Euler number correlated with increased BAG. No associations were found between genotype, hemoglobin, overt stroke history, chronic transfusion therapy, hydroxyurea use, SCI presence, or other variables and BAG. On multivariable analysis, intracranial vasculopathy (β [SE], 6.562 [1.883]; 95% CI, 2.828 to 10.296; *P* < .001) and HbS percentage (β [SE] per 1% increase, 0.089 [0.032]; 95% CI, 0.026 to 0.151; *P* = .006) remained associated with BAG in the SCA cohort (eTable 2 in Supplement 1).

Economic deprivation defined by ADI across all participants was a median (IQR) of 82.5% (55.2%-96.0%) indicating low socioeconomic status. Examining ADI as a function of BAG and by cohort, socioeconomic status was lowest in participants with the highest BAG (*H* = 13.14; *df* = 3; *P* = .004) for the control cohort. However, in the SCA cohort, ADI did not vary with BAG (*H* = 1.09; *df* = 3; *P* = .78) (Figure 1D-E).

### Association of BAG With Cognitive Outcomes

All 4 cognitive scores were impaired in participants with SCA compared to those in the control cohort, given as median (IQR): executive composite (40.7 [35.3 to 49.0] vs 50.7 [44.3 to 55.0]; *P* < .001), crystallized composite (50.0 [45.0 to 58.0] vs 57.7 [52.5 to 63.7]; *P* < .001), processing speed (47.0 [37.0 to 60.0] vs 58.0 [45.0 to 67.0]; *P* = .004), and FSIQ (92.0 [80.0 to 102.0] vs 105.0 [93.0 to 113.0]; *P* < .001) scores (eTable 1 in Supplement 1). BAG was investigated as a factor associated with cognitive function in total, control, and SCA cohorts. Multivariable analysis showed that SCA diagnosis (β [SE], −5.572 [1.561]; 95%CI, −8.659 to −2.485]; *P* < .001), higher BAG (β [SE] per 1-year increase, −0.360 [0.099]; 95% CI, −0.557 to −0.164; *P* < .001), and older age (β [SE] per 1-year increase, −0.237 [0.087]; 95% CI, −0.409 to −0.066]; *P* = .007) were associated with impaired executive function. For crystallized function, higher BAG and ADI were associated with impairment. For processing speed, higher BAG, male sex, and older age were associated with impairment. Finally, higher BAG, lower socioeconomic status, and older age were associated with FSIQ (Table 2).

**Table 2.** Imaging and Clinical Factors Associated With Cognitive Performance

	Multivariable linear regression^a^	
	β (SE) [95% CI]	*P* value
**Executive function**		
Total		
BAG, per 1-y decrease	−0.360 (0.099) [−0.557 to −0.164]	<.001
SCA vs control	−5.572 (1.561) [−8.659 to −2.485]	<.001
Age, per 1-y decrease	−0.237 (0.087) [−0.409 to −0.066]	.007
Control		
BAG, per 1-y decrease	−0.516 (0.141) [−0.799 to −0.233]	<.001
SCA		
White matter MD, per 1-mm/s^2^ decrease^b^	−104.26 (30.86) [72.74 to 162.61]	<.001
**Crystallized function**		
Total		
BAG, per 1-y decrease	−0.412 (0.029) [−0.590 to −0.235]	<.001
ADI, per 1-percentile decrease	−0.089 (0.029) [0.142 to −0.033]	.002
Control		
BAG, per 1-y decrease	−0.540 (0.136) [−0.814 to −0.267]	<.001
ADI, per 1-percentile decrease	−0.097 (0.038) [0.173 to −0.021]	.01
SCA		
NA^c^	NA^c^	NA^c^
**Processing speed**		
Total		
BAG, per 1-y decrease	−0.634 (0.162) [−0.954 to −0.314]	<.001
Sex, female vs male	9.25 (2.82) [3.67 to 14.83]	.001
Age, per 1-y decrease	−0.364 (0.156) [−0.674 to −0.055]	.02
Control		
Brain volume, per 1-mL increase^d^	123.337 (43.431) [36.263 to 210.411]	.006
SCA		
Sex, female vs male	9.854 (3.439) [3.001 to 16.71]	.005
White matter MD, per 1-mm/s^2^ increase^b^	−147.47 (52.91) [−252.93 to −42.02]	.007
**FSIQ-2 composite**		
Total		
BAG, per 1-y decrease	−0.770 (0.160) [−1.085 to −0.454]	<.001
ADI, per 1-percentile decrease	−0.141 (0.052) [−0.243 to −0.039]	.007
Control		
BAG, per 1-y decrease	−1.045 (0.228) [−1.502 to −0.589]	<.001
Age, per 1-y decrease	−0.915 (0.329) [−1.575 to −0.256]	.007
SCA		
Vasculopathy^e^	−11.62 (4.775) [−21.13 to −2.112]	.02
Brain volume, per 1-mL increase^d^	54.80 (23.14) [8.728 to 100.87]	.02

Abbreviations: ADI, national area deprivation index (first to 100th percentile, with higher values demonstrating greater deprivation and lower socioeconomic status based on participant residential address); β, unstandardized regression coefficient; BAG, brain age gap (years); MD, mean diffusivity; SCA, sickle cell anemia; SE, standard error of regression coefficient.

^a^ The neuroimaging metric demonstrating the largest effect size cognitive performance on univariate testing (eTables 3-4 in Supplement 1) was entered into the multivariable model in a stepwise approach with clinical variables, which had a univariate association *P* value of <.20. Only variables retained in the final model (*P* < .05) are shown in the table.

^b^ White matter mean diffusivity (mm^2^/s) is a metric of white matter microstructure processed from diffusion tensor imaging.

^c^ No factors were retained in the multivariable model.

^d^ Brain volume is expressed as a fraction of total intracranial volume to adjust for participant head size.

^e^ Cerebral vasculopathy was defined as a more than 50% narrowing of the distal internal carotid artery or proximal middle cerebral artery by a board-certified neuroradiologist review of the time-of-flight magnetic resonance angiography performed at the time of the brain magnetic resonance imaging scan.

### BAG vs Neuroimaging Metrics of Brain Health to Estimate Cognitive Function

While BAG was associated with cognition across the total study population, we hypothesized that alternate neuroimaging metrics reflecting age-related changes or chronic ischemia may perform better than BAG. We investigated BAG in comparison with white matter MD, normalized brain volume, and infarct volume for estimating cognition.

**Figure 3.**
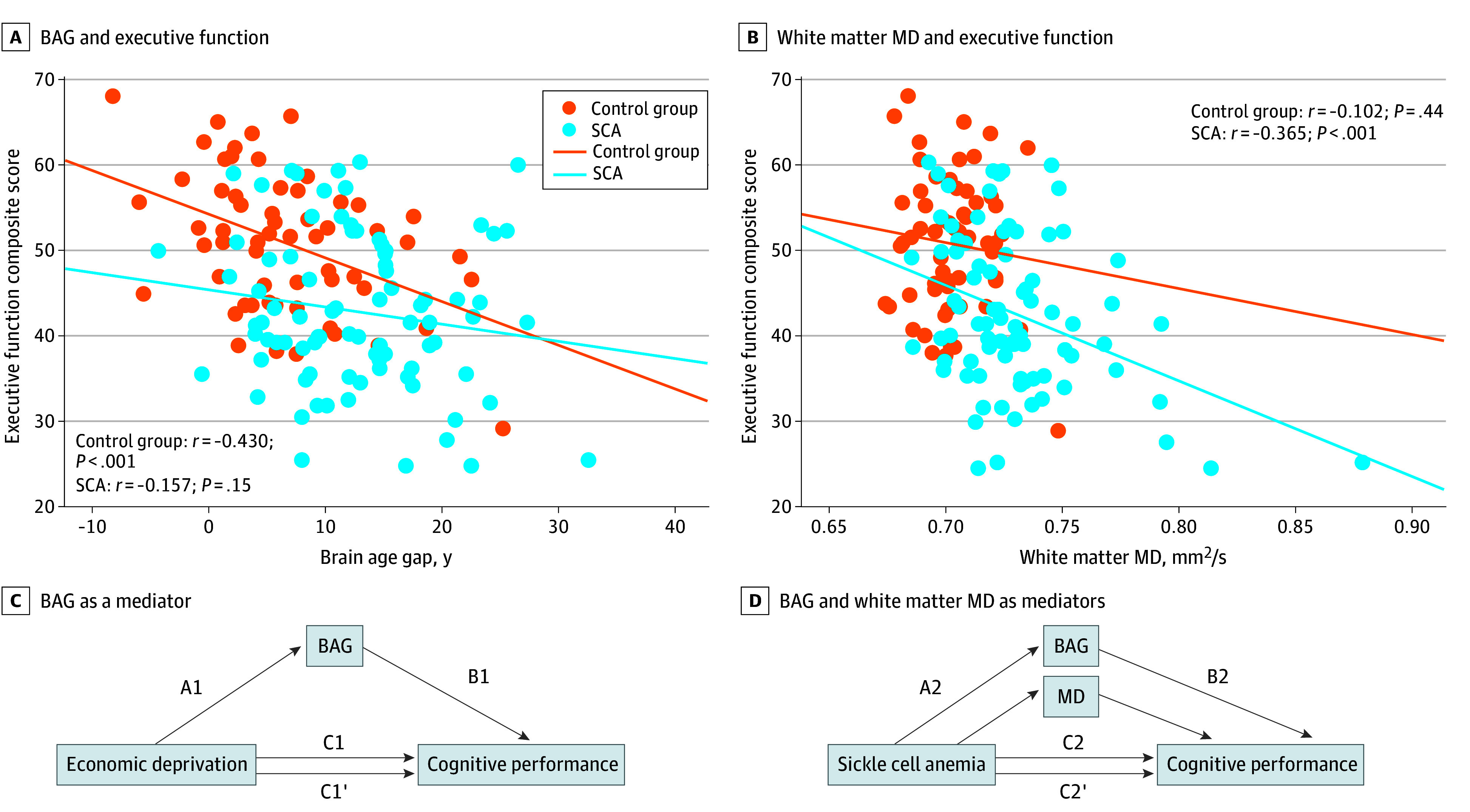
Association of Brain Age Gap (BAG) and White Matter Mean Diffusivity (MD) With Cognitive Function and Mediation of Socioeconomic Status and Sickle Cell Anemia (SCA) A, BAG was examined in association with executive function composite score in control and sickle cell anemia (SCA) cohorts. B, White matter MD was examined in association with executive function composite score in control and SCA cohorts. C, BAG as a mediator of area deprivation index (ADI)’s total effect on cognitive performance is shown, including the direct effect of ADI on cognitive performance (C1) and indirect effect of ADI on cognitive performance (C1’) after measuring the total effect of ADI on BAG (A1) and total effect of BAG on cognitive performance (B1). D, BAG and white matter MD as mediators of SCA’s total effect on cognitive performance are shown, including the direct (C2) and indirect (C2’) effects of SCA on cognitive performance after measuring the total effect of SCA on BAG (A2) and total effect of BAG on cognitive performance (B2).

In the control cohort, BAG continued to demonstrate the largest correlation with all 4 cognitive measures (executive function: *r* = −0.430; *P* = .001; crystallized function: *r* = −0.490; *P* < .001; processing speed: *r* = −0.281; *P* = .03; FSIQ composite: *r* = −0.485; *P* < .001), with the exception of processing speed, where brain volume (*r* = 0.395; *P* = .002) outperformed BAG (Figure 3A-B; eTable 3 in Supplement 1). Ischemic injury metrics, such as white matter MD and infarct volume, were not correlated with cognition in the control cohort. In multivariable analysis, BAG remained associated with executive function, crystallized function, and FSIQ. However, brain volume demonstrated a larger association than BAG with processing speed and remained associated with processing speed in multivariable regression (Table 2).

In contrast to findings in the control cohort, white matter MD, a metric that is associated with cognitive decline in adults with sporadic small vessel disease, demonstrated the largest effect size for cognitive function in the SCA cohort (executive function: *r* = −0.365; *P* = .001; crystallized function: *r* = −0.203; *P* = .07; processing speed: *r* = −0.328; *P* = .003; FSIQ composite: *r* = −0.343; *P* = .001) (Figure 3A-B; eTable 4 in Supplement 1). Infarct volume and brain volume also correlated with processing speed and FSIQ. In multivariable analysis, white matter MD remained associated with executive function and processing speed, while other metrics, including BAG were not retained. History of SCIs or overt stroke were not retained in association with cognition in the SCA cohort; however, female sex was retained in association with higher processing speed and intracranial vasculopathy was retained in association with lower FSIQ (Table 2).

### BAG as a Mediator of the Association of Socioeconomic Status With Cognition

Given its associations with ADI and cognitive performance, BAG was further examined as a mediator of the association of economic deprivation with cognition. Across the total cohort, BAG mediated the association of ADI with all 4 cognitive measures (Figure 3C; eTable 5 in Supplement 1). For estimation of executive function (β [SE] per 1-unit decrease in ADI, −0.031 [0.014]; 95% CI, −0.061 to −0.006) and processing speed (β [SE] per 1-unit decrease in ADI, −0.037 [0.018]; 95% CI, −0.076 to −0.005), BAG fully mediated the association of ADI and was otherwise a partial mediator.

### BAG and White Matter MD as Mediators of the Association of SCA With Cognition

Given that white matter MD and BAG were associated with SCA diagnosis and cognitive performance, they were examined as mediators of the association of disease with cognition. BAG mediated the association of SCA on all 4 cognitive measures (eg, FSIQ: β [SE], −3.79 [1.42]; 95% CI, −6.87 to −1.40), while white matter MD was a mediator of the association of SCA with executive function (β [SE], −2.52 [0.88]; 95% CI, −4.17 to −0.70) and FSIQ (β [SE], −4.55 [1.82]; 95% CI, −8.14 to −0.94) (Figure 3D; eTable 6 in Supplement 1).

## Discussion

Affecting up to 20 million individuals worldwide, the impact of SCA on cognitive function globally carries vast societal implications.^43^ Cognitive impairment in SCA is associated with deficits in health literacy, ability to provide self-care, and transition to adult care, as well as deficits in instrumental activities of daily living (eg, management of finances and independent living).^1^ Despite a younger chronological age in the adult population with SCA, we found an older estimated brain age and higher BAG in adults with SCA compared to healthy individuals, suggesting insufficient brain growth, premature brain aging, or both in SCA. BAG was also increased in the control compared to the reference cohort and was associated with more severe economic deprivation. Furthermore, increased BAG was associated with worse cognition across all domains. Taken together, these results indicate that individuals with SCA are living into midadulthood with signs of premature aging and that beyond the negative impact of chronic disease, economic deprivation is likely playing a critical role in brain development, aging, and, ultimately, cognitive function in reportedly healthy cohorts.^11^

A diagnosis of SCA remained independently associated with BAG across the total cohort. This finding is consistent with those of other neuroimaging investigations examining the association of SCA with brain structure and function.^44^ Clinical trials for children with SCA have focused on prevention of stroke; however, our findings and others suggest that cerebral manifestations of SCA may extend beyond stroke and that neuroprotective strategies should also use outcomes of brain structure and cognitive function.^17,45,46,47,48,49,50^ Importantly, increased BAG persisted in the SCA cohort after removal of participants who had overt stroke, intracranial vasculopathy, or large SCIs.

ADI, a metric of economic deprivation based on an individual’s residence, and lower hemoglobin were associated with BAG in the control cohort.^28,29^ Notably, levels of deprivation in SCA and control cohorts were markedly high, with median 84th and 78th percentile deprivation, respectively. The correlative nature of ADI with brain development and aging within the control cohort is supported by prior work examining the impact of poverty as measured by the income-to-needs ratio on cortical gray matter, white matter, hippocampal, and amygdala volumes in preschool children.^11^ A similar study in older adults with normal cognitive function found that ADI was associated with lower brain volumes, mediated in part by cardiovascular risk.^51^ Lower hemoglobin may be associated with older brain age due to effects of lower arterial oxygen content on the aging brain in the setting of poor nutrition or undiagnosed disease.

In contrast to the strong association between BAG and cognitive performance in healthy participants, we found that white matter MD, measured using DTI, was a stronger association of cognition across domains in the SCA cohort. This finding is consistent with non-SCA sporadic small vessel disease studies showing that white matter microstructure is one of the strongest neuroimaging predictors of progression in cognitive impairment and often more predictive than volumetric burden of white matter lesions.^52^ Global and regional brain volumes have previously been associated with cognitive performance in both children and adults with SCA.^53^ We found that whole brain volume was associated with processing speed and FSIQ on univariate analysis, yet was not retained in multivariable regression, while white matter MD was.

The wide-reaching associations of BAG across cognitive domains and its performance as a mediator of socioeconomic status and disease in the total cohort suggest that BAG, derived from a commonly acquired MRI sequence with minimal postprocessing requirements, may be a promising surrogate marker for cognitive impairment in community health care settings. Conducting and interpreting standardized cognitive assessments typically requires neuropsychologists in tertiary care centers. If a brain MRI can provide a metric of healthy vs unhealthy brain aging that predicts cognitive impairment, brain MRI could triage need for further cognitive testing in underresourced settings. Furthermore, BAG could provide a biomarker to measure the impact of neuroprotective strategies on brain health in the general population. Our data suggest that for cerebrovascular diseases such as SCA, metrics of brain health that quantify cerebral ischemic injury, such as white matter MD, may estimate cognitive outcomes as well as or better than BAG.

### Limitations

This study has several limitations. While the mediation analyses suggest that brain structure, as measured by BAG, may lie in the causal pathway from ADI to disease and cognitive function, the study is limited by its cross-sectional design, which cautions against this interpretation, and our findings would benefit from further testing using a longitudinal design. We are continuing to obtain 3-year follow-up brain MRIs in our study cohort and will complete a longitudinal analysis to investigate whether our findings are confirmed. Regarding cognitive outcomes, the NIH Toolbox adjusts for educational attainment; however, many participants with SCA would have missed school regularly for appointments and transfusions, which could have negatively impacted their cognitive outcomes. Furthermore, we expected the presence of SCIs in control and SCA cohorts to be independently associated with BAG, cognitive performance, or both; however, we did not find this result, which may be a true finding or may be due to limited sample size given that SCI presence is a dichotomous variable. DeepBrainNet was trained using a large, multicenter cohort and likely explains differences between the control cohort and reference population. Furthermore, there is a selection bias given that we purposely recruited individuals for the control cohort who would be matched to patients with SCA by age, sex, race, and socioeconomic status. With this in mind and knowing that cohorts tested in this study differ from those in the DeepBrainNet training dataset, there remained an association between BAG and ADI. This suggests that variation in brain age for these cohorts may mediate an association between socioeconomic status and cognitive function in a healthy adult cohort similar to ours. Given the relatively small sample and cross-sectional nature of our study, these findings would need to be tested in a longitudinal design of a larger cohort.

## Conclusions

Adults with SCA and those in the control cohort with greater economic deprivation demonstrated older brain age, suggestive of insufficient brain development, premature brain aging, or both. These findings suggest that brain MRI estimates of age may provide a neuroimaging biomarker of cognitive outcomes in healthy and SCA populations.

## Notes

Supplement 1.
eMethods.
**eTable 1.** Baseline characteristics and cognitive performance for subgroup with cognitive assessment **eTable 2.** Estimation of brain age gap in total, control, and SCA cohorts **eTable 3.** Neuroimaging metrics of brain health and ischemic injury in association with cognitive function in the control cohort indicating an association between structural T1 (brain age gap) and cognitive outcomes. **eTable 4.** Neuroimaging metrics of brain health and ischemic injury in association with cognitive function in the SCA cohort indicating an association between white matter microstructure and cognitive performance **eTable 5.** Indirect associations of economic deprivation with cognitive performance as mediated through brain age gap and white matter mean diffusivity **eTable 6.** Indirect associations of SCA with cognitive performance mediated through brain age gap and white matter mean diffusivity

Supplement 2.
Data Sharing Statement
