# Supplementary material for: Brain Age Modeling and Cognitive Outcomes in Young Adults With and Without Sickle Cell Anemia
Source: JAMA Netw Open. 2025 Jan 17;8(1):e2453669. doi: 10.1001/jamanetworkopen.2024.53669 (PMC11742535; doi:10.1001/jamanetworkopen.2024.53669)
Supplement: Supplement 1. — eMethods. eTable 1. Baseline characteristics and cognitive performance for subgroup with cognitive assessment eTable 2. Estimation of brain age gap in total, control, and SCA cohorts eTable 3. Neuroimaging metrics of brain health and ischemic injury in association with cognitive function in the control cohort indicating an association between structural T1 (brain age gap) and cognitive outcomes. eTable 4. Neuroimaging metrics of brain health and ischemic injury in association with cognitive function in the SCA cohort indicating an association between white matter microstructure and cognitive performance eTable 5. Indirect associations of economic deprivation with cognitive performance as mediated through brain age gap and white matter mean diffusivity eTable 6. Indirect associations of SCA with cognitive performance mediated through brain age gap and white matter mean diffusivity [file jamanetwopen-e2453669-s001.pdf]

Supplemental Online Content

Ford AL, Fellah S, Wang Y, et al. Brain age modeling and cognitive outcomes in young adults with and without sickle cell anemia. *JAMA Netw Open*. 2025;8(1):e2453669. doi:10.1001/jamanetworkopen.2024.53669

eMethods.

eTable 1. Baseline characteristics and cognitive performance for subgroup with cognitive assessment

eTable 2. Estimation of brain age gap in total, control, and SCA cohorts

eTable 3. Neuroimaging metrics of brain health and ischemic injury in association with cognitive function in the control cohort indicating an association between structural T1 (brain age gap) and cognitive outcomes.

eTable 4. Neuroimaging metrics of brain health and ischemic injury in association with cognitive function in the SCA cohort indicating an association between white matter microstructure and cognitive performance

eTable 5. Indirect associations of economic deprivation with cognitive performance as mediated through brain age gap and white matter mean diffusivity

eTable 6. Indirect associations of SCA with cognitive performance mediated through brain age gap and white matter mean diffusivity

This supplemental material has been provided by the authors to give readers additional information about their work.

© 2024 Ford AL et al. *JAMA Network Open.*

eMethods

For reasons described in the Methods, age and Euler number were included in all multivariable models. Additional clinical covariates considered for inclusion in the multivariable models for prediction of brain age gap (BAG) for the total cohort included: sex, ADI, and SCA diagnosis (control vs. SCA). Covariates considered for inclusion into the Control model were: sex, hemoglobin, ADI, and sickle cell trait status (HbAA vs. HbAS). Covariates considered for inclusion into the SCA model were: sex, ADI, sickle cell genotype, history of SCIs, history of overt stroke, chronic exchange transfusion therapy, intracranial vasculopathy, current hydroxyurea use, hemoglobin, and HbS%. Clinical covariates with p value <0.20 on univariate regression were entered into a stepwise multivariable linear model, with a p value <0.05 required for retention in the final model.

eTable 1. Baseline Characteristics and Cognitive Performance

	Healthy Control Cohort N = 60	Sickle Cell Anemia Cohort N = 87	P-value
Age (years) [a]	27.9 [25.3, 32.2]	28.9 [23.0, 36.5]	0.810
Brain Age (years) [b]	33.5 [27.9, 41.1]	43.5 [33.8, 49.7]	< 0.001
Brain Age Gap (years) [c]	5.4 [1.9, 10.1]	11.8 [7.4, 17.0]	< 0.001
**Race**			
Black American	60 (100%)	87 (100%)	1.0
**Sex**			
Male	15 (25%)	27 (31%)	0.426
Female	45 (75%)	60 (69%)	
**Cognitive Performance [d]**			
Executive Composite	50.7 [44.3, 55.0]	40.7 [35.3, 49.0]	< 0.001
Crystallized Composite	57.7 [52.5, 63.7]	50.0 [45.0, 58.0]	< 0.001
Processing Speed	58.0 [45.0, 67.0]	47.0 [37.0, 60.0]	0.004
FSIQ-2 Composite	105.0 [93.0, 113.0]	92.0 [80.0, 102.0]	< 0.001

Abbreviations: FSIQ = full scale intelligence quotient.
[a] Chronological age in years on day of MRI scan.
[b] Estimated brain age using T1 MRI scan and DeepBrainNet model.
[c] Brain age gap (BAG) in years = [Estimated brain age] – [chronological age].
[d] Executive Function Composite, Crystallized Function Composite, and Processing Speed Fully Corrected T-Score were obtained from the NIH Toolbox. The FSIQ Composite was obtained using the WASI 2nd edition.

© 2024 Ford AL et al. *JAMA Network Open.*

eTable 2. Prediction of brain age gap in total, control, and SCA cohorts

		Univariate	Multivariable Linear Regression [a]		
**Dependent Variable**	**Predictors**	**r (P-value)**	**β (SE)**	**95% Confidence Intervals**	**P-value**
**BAG, Total**	**SCA (vs. control)**	0.404 (<0.001)	5.930 (0.926)	4.105, 7.755	<0.001
	**Euler [b]**	-0.285 (<0.001)	-0.088 (0.027)	-0.141, -0.035	0.001
	**Age**	-0.137 (0.038)	-0.0.085 (0.056)	-0.195, 0.025	0.129
**BAG, Controls**	**ADI**	0.250 (0.014)	0.079 (0.028)	0.023, 0.135	0.006
	**Hb**	-0.132 (0.176)	-0.962 (0.420)	-1.796, -0.128	0.024
	**Age**	-0.008 (0.935)	-0.172 (0.104)	-0.378, 0.034	0.102
	**Euler [b]**	-0.148 (0.127)	-0.101 (0.061)	-0.222, 0.021	0.103
**BAG, SCA**	**Vasculopathy [c]**	0.309 (<0.001)	6.562 (1.883)	2.828, 10.296	<0.001
	**HbS%**	0.131 (0.149)	0.089 (0.032)	0.026, 0.151	0.006
	**Age**	-0.149 (0.099)	-0.133 (0.070)	-0.271, 0.005	0.058
	**Euler [b]**	-0.303 (<0.001)	-0.052 (0.032)	-0.116, 0.013	0.113

Abbreviations: BAG = Brain age gap; SCA = Sickle cell anemia; r = Pearson's correlation coefficient; β = unstandardized regression coefficient; SE = Standard error of regression coefficient; ADI = National area deprivation index (1-100 percentile with higher value demonstrating greater deprivation and lower socioeconomic status based on participant's residential address), Hb = hemoglobin in g/dL, HbS% = percentage of HbS on hemoglobin electrophoresis.

[a] Euler number and age at scan were entered into the model and reported in the table regardless of univariate strength of association with dependent variable given known factors which may influence BAG; subsequently, clinical variables were entered into the model stepwise with only significant, retained variables reported in the table.

[b] Euler Number, a FreeSurfer index of image quality with lower number indicating worse image quality, was adjusted for.

[c] Intracranial vasculopathy was defined as > 50% narrowing of the distal internal carotid artery or proximal middle cerebral artery by a board-certified neuroradiologist review of the time-of-flight MRA performed at time of brain MRI scan.

© 2024 Ford AL et al. *JAMA Network Open.*

eTable 3. Neuroimaging metrics of brain health and ischemic injury in association with cognitive function across domains in controls indicating a strong relationship between structural T1 in association with cognitive outcomes.

		Executive Composite	Crystallized Composite	Processing Speed	FSIQ Composite
Brain Age Gap	*r*	**-0.430**	**-0.490**	**-0.281**	**-0.485**
	P-value	**0.001**	**0.000**	**0.031**	**0.000**
White Matter Mean Diffusivity	*r*	-0.102	-0.046	-0.006	-0.168
	P-value	0.442	0.731	0.964	0.199
Infarct Volume	*r*	-0.076	-0.050	-0.028	-0.132
	P-value	0.567	0.709	0.833	0.315
Brain Volume	*r*	0.152	-0.247	**0.395**	-0.199
	P-value	0.250	0.059	**0.002**	0.128

*Raw P-values are shown and significance was retained (in bold) after correcting for multiple cognitive tests.

eTable 4. Neuroimaging metrics of brain health and ischemic injury in association with cognitive function across domains in the SCA cohort indicating a strong relationship between white matter microstructure and cognitive performance in individuals with SCA.

		Executive Composite	Crystallized Composite	Processing Speed	FSIQ Composite
Brain Age Gap	*r*	-0.157	-0.150	-0.171	-0.146
	P-value	0.151	0.171	0.118	0.177
White Matter Mean Diffusivity	*r*	**-0.365**	-0.203	**-0.328**	**-0.343**
	P-value	**0.001**	0.067	**0.003**	**0.001**
Infarct Volume	*r*	-0.091	0.115	**-0.250**	-0.149
	P-value	0.409	0.296	**0.022**	0.170
Brain Volume	*r*	0.203	0.155	**0.282**	**0.340**
	P-value	0.066	0.162	**0.010**	**0.001**

*Raw P-values are shown and significance was retained (in bold) after correcting for multiple cognitive tests.

© 2024 Ford AL et al. *JAMA Network Open.*

eTable 5. Indirect effects of economic deprivation on cognitive performance as mediated through brain age gap and white matter mean diffusivity

Dependent Variable	Indirect effect of SES (ADI) as mediated by: Brain Age Gap				Indirect effect of SES (ADI) as mediated by: White Matter MD			
	**Effect**	**SE**	**Lower**	**Upper**	**Effect**	**SE**	**Lower**	**Upper**
**Executive Function [a]**	-.031 [b, c]	.014	-.061	-.006	-.024	.015	-.056	.003
**Crystallized Function [a]**	-.026 [b]	.011	-.051	-.006	-.013	.009	-.034	.002
**Processing Speed [d]**	-.037 [b, c]	.018	-.076	-.005	-.022	.016	-.057	.005
**FSIQ-2 [a]**	-.045 [b]	.021	-.089	-.007	-.034	.024	-.092	.005

Abbreviations: SES = Socioeconomic status; ADI = National area deprivation index (1-100 percentile with higher value demonstrating greater deprivation and lower socioeconomic status based on participant's residential address); BAG = Brain age gap; MD = mean diffusivity; SE = Standard error; Lower, Upper = limits of 95% confidence intervals.

[a] Model adjusted by age

[b] $P < 0.05$

[c] Full mediation of independent variable by BAG. Unless indicated, mediation is partial.

[d] Model adjusted by age and sex

eTable 6. Indirect effects of sickle cell anemia on cognitive performance as mediated through brain age gap and white matter mean diffusivity

Dependent Variable	Indirect Effect of SCA as mediated by: Brain Age Gap				Indirect Effect of SCA as mediated by: White Matter MD			
	**Effect**	**SE**	**Lower**	**Upper**	**Effect**	**SE**	**Lower**	**Upper**
**Executive Function [a]**	-2.14 [b]	.801	-3.85	-.726	-2.52 [b]	.879	-4.165	-.696
**Crystallized Function [a]**	-2.22 [b]	.757	-3.87	-.819	-1.32	.788	-2.866	.275
**Processing Speed [c]**	-3.31 [d, e]	1.52	-6.61	-.728	-3.22	1.591	-6.054	.215
**FSIQ-2 [a]**	-3.79 [b]	1.42	-6.87	-1.40	-4.55 [d]	1.816	-8.139	-.942

Abbreviations: SCA= sickle cell anemia; BAG = Brain age gap; MD = mean diffusivity; SE = Standard error; Lower, Upper = limits of 95% confidence intervals.

[a] Model adjusted by age

[b] $P < 0.01$

[c] Model adjusted by age and sex

[d] $P < 0.05$

[e] Full mediation of independent variable by BAG. Unless indicated, mediation is partial.

© 2024 Ford AL et al. *JAMA Network Open.*
