# Supplementary material for: Brain Age Modeling and Cognitive Outcomes in Young Adults With and Without Sickle Cell Anemia
Source: JAMA Netw Open. 2025 Jan 17;8(1):e2453669. doi: 10.1001/jamanetworkopen.2024.53669 (PMC11742535; doi:10.1001/jamanetworkopen.2024.53669)
Supplement: Supplement 2. — Data Sharing Statement [file jamanetwopen-e2453669-s002.pdf]

Data Sharing Statement

Ford. Brain Age Modeling and Cognitive Outcomes in Young Adults With and Without Sickle Cell Anemia. *JAMA Netw Open*. Published online November 8, 2025. doi:10.1001/jamanetworkopen.2024.53669

Data
Data available: Yes
Data types: Deidentified participant data, Data dictionary **How to access data:** forda@wustl.edu
When available: With publication

Supporting Documents
Document types: None

Additional Information
Who can access the data: Researchers whose proposed use of the data has been approved
Types of analyses: For a specified purpose
Mechanisms of data availability: without investigator support and after approval of a proposal
